# Epigenetic inactivation of the 5-methylcytosine RNA methyltransferase *NSUN7* is associated with clinical outcome and therapeutic vulnerability in liver cancer

**DOI:** 10.1186/s12943-023-01785-z

**Published:** 2023-05-12

**Authors:** Vanessa Ortiz-Barahona, Marta Soler, Veronica Davalos, Carlos A. García-Prieto, Maxime Janin, Fernando Setien, Irene Fernández-Rebollo, Joan J. Bech-Serra, Carolina De La Torre, Sonia Guil, Alberto Villanueva, Pei-Hong Zhang, Li Yang, Marco Guarnacci, Ulrike Schumann, Thomas Preiss, Ugne Balaseviciute, Robert Montal, Josep M. Llovet, Manel Esteller

**Affiliations:** 1 Cancer Epigenetics Group, Josep Carreras Leukaemia Research Institute, Badalona, Barcelona, Catalonia 08916 Spain; 2 Life Sciences Department, Barcelona Supercomputing Center (BSC), Barcelona, Catalonia Spain; 3 Proteomics Unit, Josep Carreras Leukaemia Research Institute, Badalona, Barcelona, Catalonia 08916 Spain; 4 Regulatory RNA and Chromatin Group, Josep Carreras Leukaemia Research Institute, Badalona, Barcelona, Catalonia 08916 Spain; 5 Germans Trias i Pujol Health Science Research Institute, Barcelona, Catalonia Spain; 6 Chemoresistance and Predictive Factors Group, Program Against Cancer Therapeutic Resistance (ProCURE), Catalan Institute of Oncology (ICO), Oncobell Program, Bellvitge Biomedical Research Institute (IDIBELL), L’Hospitalet del Llobregat, Barcelona, Catalonia Spain; 7 Shanghai Institute of Nutrition and Health, University of Chinese Academy of Sciences, Chinese Academy of Sciences, Shanghai, China; 8 Center for Molecular Medicine, Children’s Hospital, Fudan University and Shanghai Key Laboratory of Medical Epigenetics, International Laboratory of Medical Epigenetics and Metabolism, Institutes of Biomedical Sciences, Fudan University, Shanghai, China; 9 Shine-Dalgarno Centre for RNA Innovation, Australian National University, Canberra, Australia; 10 Victor Chang Cardiac Research Institute, Darlinghurst (Sydney), Queensland, NSW 2010 Australia; 11 Institut d’Investigacions Biomèdiques August Pi i Sunyer (IDIBAPS), Hospital Clínic, University of Barcelona, Catalonia, Spain; 12 Hospital Arnau de Vilanova, IRBLleida, University of Lleida (UdL), Catalonia, Spain; 13 ICAHN School of Medicine at Mount Sinai, New York, NY USA; 14 Institució Catalana de Recerca i Estudis Avançats (ICREA), Barcelona, Catalonia 08010 Spain; 15 Centro de Investigacion Biomedica en Red Cancer, Madrid, 28029 Spain; 16 Physiological Sciences Department, School of Medicine and Health Sciences, University of Barcelona, Barcelona, Catalonia 08907 Spain

**Keywords:** RNA modifications, 5-methylcytosine RNA methyltransferase, NSUN7, Epigenetics, DNA methylation, Liver cancer

## Abstract

**Background:**

RNA modifications are important regulators of transcript activity and an increasingly emerging body of data suggests that the epitranscriptome and its associated enzymes are altered in human tumors.

**Methods:**

Combining data mining and conventional experimental procedures, *NSUN7* methylation and expression status was assessed in liver cancer cell lines and primary tumors. Loss-of-function and transfection-mediated recovery experiments coupled with RNA bisulfite sequencing and proteomics determined the activity of NSUN7 in downstream targets and drug sensitivity.

**Results:**

In this study, the initial screening for genetic and epigenetic defects of 5-methylcytosine RNA methyltransferases in transformed cell lines, identified that the NOL1/NOP2/Sun domain family member 7 (*NSUN7*) undergoes promoter CpG island hypermethylation-associated with transcriptional silencing in a cancer-specific manner. *NSUN7* epigenetic inactivation was common in liver malignant cells and we coupled bisulfite conversion of cellular RNA with next-generation sequencing (bsRNA-seq) to find the RNA targets of this poorly characterized putative RNA methyltransferase. Using knock-out and restoration-of-function models, we observed that the mRNA of the coiled-coil domain containing 9B (*CCDC9B*) gene required NSUN7-mediated methylation for transcript stability. Most importantly, proteomic analyses determined that CCDC9B loss impaired protein levels of its partner, the MYC-regulator Influenza Virus NS1A Binding Protein (IVNS1ABP), creating sensitivity to bromodomain inhibitors in liver cancer cells exhibiting *NSUN7* epigenetic silencing. The DNA methylation-associated loss of *NSUN7* was also observed in primary liver tumors where it was associated with poor overall survival. Interestingly, *NSUN7* unmethylated status was enriched in the immune active subclass of liver tumors.

**Conclusion:**

The 5-methylcytosine RNA methyltransferase *NSUN7* undergoes epigenetic inactivation in liver cancer that prevents correct mRNA methylation. Furthermore, *NSUN7* DNA methylation-associated silencing is associated with clinical outcome and distinct therapeutic vulnerability.

**Supplementary Information:**

The online version contains supplementary material available at 10.1186/s12943-023-01785-z.

## Introduction

Over 100 distinctly modified nucleotides have been reported in RNA to be involved in the regulation of non-coding and coding transcripts. This landscape of RNA modifications, denominated the epitranscriptome, is a critical layer which defines cellular activity. In this regard, the observed aberrant expression of RNAs and proteins in transformed cells could also be related to alterations in the patterns of these RNA chemical marks. They could, in turn result from defects to the enzymes and binding proteins, that act as writers, erasers and readers of the epitranscriptome [1, 2]. The most abundant and studied modification of RNA is the methylation of adenosine (A) in the form of m^6^A. However, RNA transcripts also exhibit 5-methylcytosine (m^5^C) marks, which is also known to be present on the DNA [3–6]. Although m^5^C in RNA is widely present in distinct species [7] and its existence in messenger RNA (mRNA) has been known since the 1970s [8, 9], its functional activities are still largely unknown. The m^5^C modification occurs both in coding transcripts, but also in transfer RNA (tRNA), ribosomal RNA (rRNA) and other non-coding RNA transcripts [3–5]. Multiple effects of the m^5^C marks in these transcripts are starting to emerge, such as the regulation of RNA stability, processing, translation and export [10–12]. The target sites and levels of m^5^C depends on a complex set of eight specifically devoted 5-methylcytosine RNA methyltransferases in humans, constituted by the tRNA aspartic acid MTase 1 (TRDMT1, also known as DNMT2) and by seven proteins of the NOP2/Sun domain family (NSUN1-7) [13]. In recent years, the role of these 5-methylcytosine RNA methyltransferases in cancer has started to be unveiled. In this regard, altered expression of the tRNA 5-methylcytosine RNA methyltransferase NSUN2 is commonly observed in human malignancies [14] and its deletion in mouse skin tumors increases self-renewal capacity [15]; whereas epigenetic loss of the rRNA 5-methylcytosine RNA methyltransferase *NSUN5* occurs in gliomas driving a stress adaptive translational program [16].

Herein, we provide another twist to the connection between the epigenome and the epitranscriptome, by showing that cancer cells undergo promoter CpG island hypermethylation-associated transcriptional silencing of the 5-methylcytosine RNA methyltransferase *NSUN7*. Focusing on one of the most targeted tumor types by this epigenetic disruption, liver cancer, we characterized the completely undescribed activity of NSUN7 as a writer of the m^5^C mark in mRNA. Importantly, we found that NSUN7 epigenetic loss destabilized the coiled-coil domain containing 9B (*CCDC9B*) mRNA by preventing the correct methylation of the transcript, also inducing upregulation of the CCDC9B protein partner IVNS1ABP, involved in MYC signaling. Strikingly, this cascade of events renders liver cancer cells more sensitive towards the growth-inhibitory actions of bromodomain inhibitors. Interestingly, we observed that, in the clinical setting, the presence of *NSUN7* epigenetic silencing in primary liver tumors was associated with poor clinical outcome. Interestingly, the *NSUN7* unmethylated cases exhibited an immune active signature; suggesting that this subset of patients could be particularly amenable for the use of immune checkpoint inhibitors.

## Methods

### Human cell lines and tissues

Human hepatocellular carcinoma (HCC) cell lines were purchased from the ATCC (HEP3B2-1-7, SNU-475, SNU-387 and SNU-423); the JCRB Cell Bank (HUH-7) and the SNU-398 cell line was kindly provided by Dr.Josep M. Llovet (Icahn School of Medicine at Mount Sinai, New York, USA, and IDIBAPS-Hospital Clínic, Barcelona, Spain). Cell lines were cultured in Roswell Park Memorial Institute (RPMI) medium supplemented with 10% (v/v) fetal bovine serum (FBS) (Innovative), and 1% v/v penicillin/streptomycin (Gibco).

### DNA extraction

DNA was extracted from cell pellets firstly by overnight incubation with lysis buffer (10 mM Tris pH 8, 5 mM EDTA, 100 mM NaCl, 1% SDS, 10 mg/mL proteinase K) at 37 °C. Then, 5 M NaCl was added to cell lysates, and samples were centrifuged 15 min at 4000 rpm. Upper phases were collected, and DNA was precipitated by isopropanol addition. DNA was washed with 70% ethanol, air-dried and resuspended in water. DNA concentration was measured by Nanodrop.

### DNA bisulfite conversion

Genomic DNA was bisulfite converted using the EZ DNA Methylation-Gold kit (Zymo Research) following manufacturer’s instructions and as previously described [16]. Bisulfite treatment converts unmethylated cytosines into uracil, whereas methylated cytosines remain unchanged.

### DNA methylation microarrays

Genome-wide promoter methylation status was determined using the HumanMethylation450 Beadchip (450 K) (Illumina). Bisulfite converted DNA was used for hybridization onto the beadchip. Raw fluorescence intensity values were normalized with Illumina Genome Studio software, and used to calculate DNA methylation levels (β-values).

### Bisulfite sequencing PCR (BSP)

DNA methylation status of 5’-end promoter-associated CpG island of candidate genes was analyzed by bisulfite sequencing. Bisulfite modification of DNA was performed as explained above and as previously described [16]. Promoter regions were obtained from the UCSC Genome Browser, and CpG islands were identified *in silico* using Methyl Primer Express v1.0 (Applied Biosystems). Primers encompassing the transcription start site (TSS)-associated CpGs were designed using the same program. Primer sequences can be found in **Key Resources Table**. A specific region of the promoter of interest was then PCR amplified, cloned into pGEM®-T Easy vector (Promega), and transformed into competent bacteria. Bacterial plasmid DNA was extracted and purified. A minimum of eight single clones were submitted to sequencing PCR using BigDye Terminator V3.1 cycle sequencing kit (Applied Biosystems) and sequenced in a 3730 DNA Analyzer (Applied Biosystems). Reads were aligned using the BioEdit software, and the methylation status of the promoter-associated island region was represented using the BSMap software.

### Expression analyses

RNA expression was assessed by real time quantitative reverse transcription PCR (qRT-PCR) experiments. Total RNA was first extracted from cell pellets using the SimplyRNA kit (Promega) on a Maxwell RSC device (Promega). 2 µg of total RNA were reverse-transcribed using GoScript Reverse Transcription system (Promega) following manufacturer’s instructions. Quantitative PCR was performed using SYBR® Green PCR Master Mix (Applied Biosystems) in a QuantStudio 5 Real-Time PCR System (ThermoFisher). GAPDH was used as the housekeeping gene to enable normalization. Reactivation treatments with the demethylating agent 5-aza-2′-deoxycytidine (5-Aza-dC; Sigma) were performed at 0.5 µM and 1 µM for 72 h. RNA extraction and qRT-PCR were carried out as described before. Protein expression was analyzed by western blotting. Total protein extracts were obtained using RIPA buffer (50 mM Tris pH 7.5, 150 mM NaCl, 1 mM EDTA and EGTA, 1% NP40, 0.5% sodium deoxycholate, 0.1% SDS) containing protease inhibitor cocktail cOmplete™ (Roche). Protein levels were quantified using Pierce™ BCA protein assay kit (Thermo Scientific) following manufacturer’s recommendations. Proteins were resolved on SDS-polyacrylamide gels, transferred to nitrocellulose membranes, and blotted against specific antibodies. The qRT-PCR primers and primary antibodies used are listed in the **Key Resources Table**.

### Cellular overexpression model

The cDNA sequence of *NSUN7* was cloned into the pLVX-IRES-ZsGreen1 expression plasmid (Clontech) and the mutated cDNA version was cloned into the pLVX-IREStdTomato vector (Addgene). A normal peripheral blood mononuclear cells (PBMCs) RNA sample, from a healthy donor, was reverse-transcribed and used to amplify *NSUN7* coding sequence. Specific adapter primers were designed to add restriction sites for XhoI and NotI to 5’ and 3’ ends of *NSUN7* cDNA sequence. This construct was used as a template to incorporate a specific point mutation at the active site (C439V) by a PCR phusion strategy. Oligonucleotide sequences are listed in the **Key Resources Table**. Lentiviruses containing the aforementioned constructs (*NSUN7* wild-type or mutated), or the empty vectors (EV) were produced by co-transfecting HEK-293T cells with psPAX2 (Addgene) and pMD2.G (Addgene), using jetPRIME® Transfection Reagent (Polyplus Transfection). The *NSUN7*-silenced SNU-423 cell line was lentiviral-transduced with the different constructs, GFP or Tomato-FACS sorted and maintained in culture as already stated.

### CRISPR-mediated depletion model

The NSUN7-expressing HCC cell line HEP3B2-1-7 was depleted of its expression by using a CRISPR-mediated knock out strategy. Three different guide RNAs (gRNAs) were designed to target the Cas9 machinery to different *NSUN7* exons within the functional domain. For each gRNA, a pair of oligos were purchased (Sigma), annealed and cloned into the pSpCas9-2 A-GFP plasmid (Clontech). HEP3B2-1-7 cells were transiently transfected with the pool of gRNAs using jetPRIME® Transfection Reagent (Polyplus Transfection), and green fluorescent cells were sorted by FACS and subjected to clonal proliferation. *NSUN7* knock-out (KO) was assessed by qRT-PCR and western blot.

### RNA bisulfite sequencing

Total RNA was bisulfite converted using the Methylamp RNA Bisulfite Conversion Kit (EpiGentek) following a modified version of the manufacturer’s protocol, tripling the cycles stated, as previously described [16]. Bisulfite-modified RNA was reverse-transcribed using GoScript Reverse Transcription system (Promega) following manufacturer’s instructions, with random hexamers. PCR primers for the sequences of interest, containing the potentially methylated cytosines, were designed with Primer3web (https://primer3.ut.ee/), aiming for smaller amplicon sizes in order to reduce amplification of unconverted cytosines, due to strong secondary structure. Primer sequences are available in **Key Resources Table**. PCR amplicons were purified and ligated into pGEM®-T Easy vector (Promega), transformed and sequenced as described previously in Bisulfite genomic sequencing section. Sanger sequencing allows discriminating between methylated and non-methylated cytosines, by detecting C-to-T conversions of non-methylated Cs. However, bisulfite ions are refractory to react with double-stranded molecules, which makes complete denaturation of complex RNA secondary structures crucial, in order to achieve complete conversion of all non-methylated cytosines, as incomplete conversion results in false positives. In order to identify false positives, multiple clones (n > 10) of each cell line sample were sequenced, and we considered unconverted cytosines all cytosines whose methylation status was not consistently placed across all clones. These are the named “clones with incomplete bisulfite conversion” that are discarded in our targeted bisulfite sequencing of single-clones, as it is commonly standard in the field [16].

### Transcriptome-wide bisulfite RNA sequencing (bsRNA-seq)

Two independent biological replicates from EV- and *NSUN7*-transfected SNU-423 cells were used. Total RNA from samples was isolated using the SimplyRNA Maxwell kit, DNA was depleted with Turbo DNase (Ambion), phenol/chloroform extracted and ethanol precipitated. RNA quality was estimated on a Bioanalyzer total RNA Nano Chip (Agilent). In order to evaluate the bisulfite conversion efficiency between samples, 2 ng of two Renilla Luciferase in vitro transcripts were spiked in 10 µg of each RNA replicate. Ribosomal RNA (rRNA) was reduced with the Ribo-Zero rRNA Removal Kit (Illumina) prior to bisulfite conversion, and the efficiency of rRNA depletion was checked using a Bioanalyzer total RNA Pico Chip (Agilent). Bisulfite conversion was carried out as described before [6]. Libraries were prepared according to Illumina TruSeq Stranded Total RNA protocol and sequenced on an Illumina Hiseq2500, in paired end mode and 2 × 100-bp length using the Illumina HiSeq Rapid SBS kit v2. The quality of the raw fastq files was checked with the FastQC software, and low-quality bases and adaptor sequences were removed using Trimmomatic (v0.36). Unmapped reads were mapped to the appropriate converted reference (hg38 build) using the meRanGh tool in MeRanTK. For m^5^C site calling, the following criteria were applied as previously described [6]: both replicates should have a coverage of at least 30 reads (’30RC’), a minimal depth of at least five cytosines (‘5 C’), at least 80% of bases identified are cytosine or thymidine (‘80CT’) and average non-conversion level across replicates of at least 10% (‘10MM’). If a site passed the aforementioned filters in only one replicate, then a stricter set of filters was applied (‘30RC’, ‘7 C’, ‘80CT’ and ‘10MM’). The used bioinformatic scripts are available at GitHub (https://github.com/YangLab/bsRNA-seq-m5C) and they have been previously validated [6]. Differential methylation analysis between conditions (EV- vs *NSUN7*-transfected) using Fisher’s exact test was performed. Cytosines differentially methylated between conditions were defined as those with a multiple correction adjusted p-value < 0.01 and a methylation difference over 0.1.

### RNA stability determination by actinomycin D chase assay

Treatments with the transcription inhibitor Actinomycin D (Sigma) were performed at 1 µg/ml for 24 h. At the indicated timepoints, cells were pelleted, and RNA was extracted and reverse-transcribed as explained above. Quantitative PCR was performed using SYBR® Green PCR Master Mix (Applied Biosystems) in a QuantStudio 5 Real-Time PCR System (ThermoFisher). GAPDH was used as the housekeeping gene to enable normalization.

### Immunoprecipitation and mass spectrometry-based protein-protein interactions (IP-MS/MS)

Protein extracts from HEP3B2-1-7 Empty Vector (EV)- and *CCDC9B*-FLAG-transfected cells were immunoprecipitated with Anti-Flag M2 Magnetic Beads (Sigma) in 1X TBS buffer according to manufacturer’s instructions. Beads were then washed five times with 1X TBS buffer. Prior to digestion, immunoprecipitation efficiency was assessed by boiling and loading 10% of beads with 1X Laemmli buffer on a polyacrylamide gel. The remaining 90% of beads were washed with 100mM Tris and reduced and alkylated with DTT and CAA in 6 M Urea 100mM Tris. Proteins were overnight digested with 1 ug of trypsin at 30 °C. Samples were desalted using PolyLC C18 pipette tips, and tryptic peptides were reconstituted with 3% acetonitrile and 0.1% formic acid aqueous solution. Peptides were separated using an Evosep EV1106 column (150 μm × 150 mm, 1.9 μm) (Evosep) at a flow rate of 500nL/min with an 88 min run and fitted on an Orbitrap Eclipse™ Tribrid (Thermo Scientific), using higher energy collisional dissociation (HCD) fragmentation. The mass spectrometer was operated in positive mode with spray voltage set at 1.9 kV and source temperature at 275 °C. The mass spectrometer was operated in a data-dependent acquisition (DDA) mode, with full MS scans over a mass range of m/z 350–1,400. Survey MS scans were acquired in the Orbitrap with the resolution m/z range (350-1,400 a.m.u), the highest charge state ions per scan were fragmented in the HCD (28% Collision Energy) and detected in the Orbitrap (MS1 120k, and MS2 30k) resolution. All data were acquired with Xcalibur software (v4.2.28.14) (Thermo Scientific). Raw files were analyzed with the MaxQuant software (v.2.0.1.0) using the built-in search engine Andromeda to search against the Swissprot Human database downloaded from UniprotKB website in March 2, 2021 (20,394 entries). The final list of identified peptides and proteins were filtered by using a 1% False Discovery Rate (FDR) both at peptide and protein level. To enhance the identification of proteins the “match between runs” option was selected. The statistical analysis of the protein-protein interactions found in our experimental conditions was performed with the Significance Analysis of the INTeractome (SAINT) algorithm [17]. Previously, we created the input files for SAINT using the MaxQuant output files called “msms.txt” and “proteinGroups.txt” and the scripts “PSM_Calculation.R” and “SAINTFilesCreator.R” (both included in the proteomeXchange repository with accession number PXD038422). The used bioinformatic scripts for the interactome study are available at GitHub (https://github.com/mesteller-bioinfolab/NSUN7).

### Tandem mass tag (TMT)-based MS proteomics

Three independent biological replicates from EV- and *NSUN7*-transfected SNU-423 cells were used. Cell pellets were lysed in Urea-based buffer (6 M Urea, 100 mM Tris-HCl pH 7.5 and protease and phosphatase inhibitor cocktails) on a Bioruptor sonicator. Lysates were incubated on ice for 30 min followed by centrifugation at 20,000 g to remove insoluble debris. Proteins were overnight precipitated with 100% w/v TCA, and then pellets were washed with chilled acetone for 30 min. Samples were then centrifuged and resuspended with 6 M Urea Tris 0,1 M and quantified by DCTM Protein Assay (BIO-RAD) kit. Protein digestion was carried out with Lys-C and Trypsin overnight at 30 °C. Enzymatic reaction was stopped with formic acid (10% (v/v) final concentration). 200 ug of digested peptides were acidified by the addition of formic acid and purified using reversed-phase C18 cartridges (SepPak Classic, 500 mg sorbent, Waters) according to manufacturer’s instructions. Peptides were reconstituted in 50 mM HEPES (pH 8.5) and TMT16-plex reagents (ThermoFisher) were added from stocks dissolved in 100% anhydrous ACN at a protein:TMT ratio of 1:1.5 (w/w), and the peptide–TMT mixture was incubated for 1 h at 25 °C and 400 rpm. Labelled peptides were desalted using RP solid-phase extraction cartridges (Waters), fractionated with Zorbax Extent-C18 (2.1 × 150 mm 3.5 μm 300 A) into 96 fractions, and pooled into 48 fractions. Peptides were separated using an Evosep EV1106 column (150 μm × 150 mm, 1.9 μm) (Evosep) at a flow rate of 500nL/min with an 88 min run. The column outlet was directly connected to an EASY-Spray source (Thermo) fitted on an Orbitrap Eclipse™ Tribrid (Thermo Scientific). The mass spectrometer was operated in a data-dependent acquisition (DDA) mode. In each data collection cycle, one full MS scan (375–1500 m/z) was acquired in the Orbitrap (1.2 × 105 resolution setting and automatic gain control (AGC) of 2 × 105). The following MS2-MS3 analysis was conducted with a top speed approach. The most abundant ions were selected for fragmentation by collision induced dissociation (CID). CID was performed with a collision energy of 35%, 0.25 activation Q, an AGC target of 1 × 104, an isolation window of 0.7 Da, a maximum ion accumulation time of 50 ms and turbo ion scan rate. Previously analyzed precursor ions were dynamically excluded for 30 s. For the MS3 analyses for TMT quantification, multiple fragment ions from the previous MS2 scan (SPS ions) were co-selected and fragmented by HCD using a 65% collision energy and a precursor isolation window of 2 Da. Reporter ions were detected using the Orbitrap with a resolution of 30,000, an AGC of 1 × 105 and a maximum ion accumulation time of 120 ms. Raw files were analyzed with PEAKS X + software to search against the Swissprot Human database downloaded from UniprotKB website (March 2, 2021; 20,394 entries). The final list of peptides and proteins were filtered at 1% FDR and only proteins represented by more than one peptide were considered as candidate targets. The intensities of the proteins included in the final list were log2-transformed. Then, the proteins were filtered to obtain only those with more than 75% of valid intensity values in each experimental condition. The resulting list was then imputed using the ‘predictive mean matching’ algorithm included in the “impute” R package. The dataset was subsequently normalized by median-centering the intensities and processed with the R packages “limma” and “DEqMS” to look for significant proteins with statistical significance. The script including all the details has been uploaded into the proteomeXchange repository with accession number PXD038418. The used bioinformatic scripts for the proteomics studies are available at GitHub (https://github.com/mesteller-bioinfolab/NSUN7).

### Cell viability

For in vitro IC_50_ studies, 5000 cells per well were seeded in 96-well plates, and maintained overnight to allow cell attachment prior to drug exposure. Bromodomain inhibitors used in this study were JQ1 (ApexBio; Cat. No. A1910), i-BET (i-BET 151; Tocris Bioscience; Cat. No. 4650) and PFI-1 (Tocris Bioscience; Cat. No. 4445/10). Cell viability was assessed by the sulforhodamine B (SRB) assay, 72 h after drug treatment. Briefly, cells were fixed with 10% trichloroacetic acid, washed twice with distilled water and stained with 0.4% SRB in 1% acetic acid for 30 min. SRB was then solubilized in 10mM Tris-HCl pH 10.0 and 540 nm-optical densities were determined using a microplate reader (Perkin Elmer Viktor 3). Data was analyzed with GraphPad Prism 5 software.

### Molecular and clinical data

Gene mutation, copy number variation (CNV), DNA methylation and mRNA expression data in cell lines were obtained from COSMIC cell line database. For The Cancer Genome Atlas (TCGA) set of HCC patients (TCGA-LIHC), DNA methylation (status of *NSUN7* promoter CpG site cg01143804), mRNA expression and clinical data were obtained from the TCGA Data Portal.

### Statistical analysis

The associations between variables were assessed by Fisher’s exact test, Unpaired t-test, or Spearman correlation as appropriate. Kaplan-Meier plots and log-rank test were used to estimate Overall Survival (OS). Univariate and multivariate Cox regression analysis were performed calculating the hazard ratio with a 95% of confidence interval. Statistical analyses were carried out by using SPSS (Armonk, NY) and GraphPad Prism 5 (La Jolla, CA). P values lower than 0.05 were considered statistically significant.

## Results

### DNA Methylation-Associated Transcriptional Inactivation of the 5-Methylcytosine RNA Methyltransferase ***NSUN7*** in Liver Cancer

**Fig. 1:**
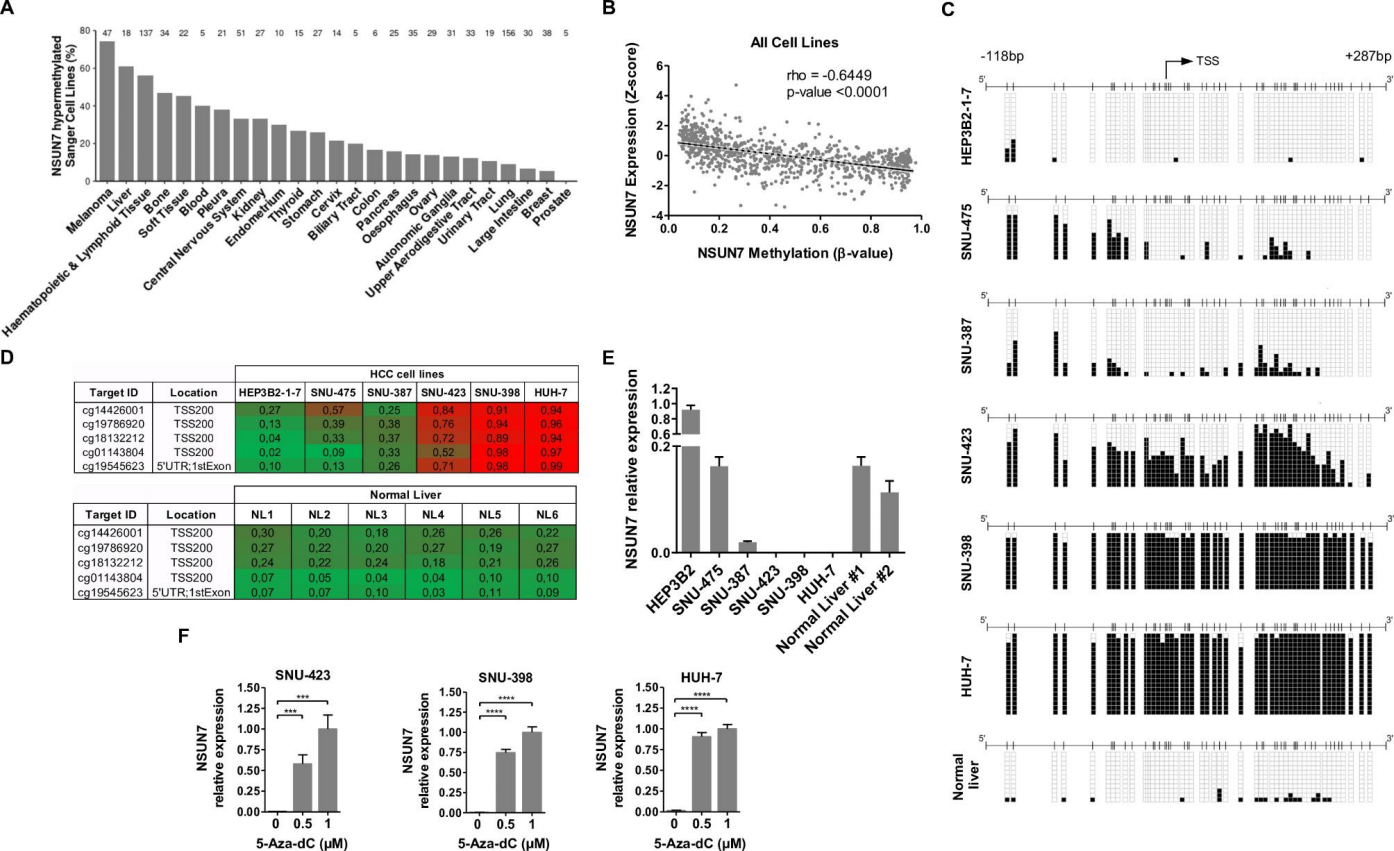
Transcriptional silencing of *NSUN7* by promoter CpG island hypermethylation in cancer cell lines. (**A**) Percentage of human cancer cell lines from the Sanger panel, classified by primary tumor site, with a hypermethylated *NSUN7* promoter. Total number of cell lines is shown on top of each bar. (**B**) Correlation analysis between *NSUN7* promoter methylation (mean β-value) and *NSUN7* transcript expression (Z-score) in cancer cell lines. Spearman’s rank correlation test with its p-value and the associated rho coefficient are shown. (**C**) Bisulfite genomic sequencing of *NSUN7*-promoter CpG Island in HCC cell lines, plus a normal liver sample. CpG dinucleotides are represented as short vertical lines, *NSUN7* TSS is indicated by a black arrow, and unmethylated or methylated cytosines are represented as white or black squares, respectively. Single clones are shown for each sample (n > 10). (**D**) Absolute methylation β-values of *NSUN7* promoter-associated CpGs analyzed by the 450 K DNA methylation microarray. Green, unmethylated; red, methylated. Data from the studied HCC cell lines, and six normal liver samples are shown. (**E**) *NSUN7* expression in the HCC cells and two normal livers at the RNA level, analyzed by real-time PCR. (**F**) *NSUN7* transcript expression is restored in the three *NSUN7* hypermethylated cell lines (SNU-423, SNU-398 and HUH-7) by treatment with the demethylating agent 5-aza-2′-deoxycytidine (5-Aza-dC). RNA expression data shown represent the mean ± S.D. of biological triplicates, and p-values were calculated by a Student’s T test. ***p-value < 0.001, ****p-value < 0.0001

To characterize possible genetic and epigenetic alterations in the RNA cytosine methyltransferases *NSUN2*, *NSUN3*, *NSUN6* and *NSUN7* in tumors, we first studied *in silico* a set of 1001 human cancer cell lines in which we had obtained the exome, transcriptome, gene copy number and DNA methylation landscapes [18]. The data mining analyses did not show the occurrence of *NSUN2*, *NSUN3*, *NSUN6* and *NSUN7* mutations, deletions or amplifications in the cell line panel (**Dataset S1**). Although DNA sequence and genomic aberrations in the aforementioned genes were not observed, promoter CpG island hypermethylation-associated transcriptional silencing is another alternative to accomplish loss of gene activity in transformed cells [19, 20]. *NSUN2*, *NSUN3* and *NSUN6* 5’-end associated CpG islands were unmethylated in all the cell lines of the collection (**Dataset S1A,B,C**). However, *NSUN7* promoter CpG island was methylated among different cancer types (**Dataset S1D**, Fig. 1A), being the three most often targeted sites in melanoma (35 of 47, 74.5%), liver cancer (11 of 18, 61.1%) and hematological malignancies (77 of 137, 56.2%). Data-mining of the available expression profiles of the cancer cell lines [18] showed that *NSUN7* methylation was associated with RNA downregulation (Fig. 1B). Due to our long-standing interest in liver cancer and many collaborative research projects in this field [21–23], we decided to focus our study of NSUN7 in this tumor type, a leading cause of cancer mortality worldwide, accounting for more than 700,000 deaths each year [24].

Following the above described biocomputational observation of *NSUN7* CpG island hypermethylation-associated silencing in cancer cell lines, we experimentally characterized this phenomenon in liver tumor cell lines. We carried out the bisulfite genomic sequencing of multiple clones in the liver cancer cell lines HEP3B2-1-7, SNU-475, SNU-387, SNU-423, SNU-398 and HUH-7 utilizing primers that encompassed the *NSUN7* transcription start site located in the 5’-end CpG island. We observed dense hypermethylation of the *NSUN7*-associated promoter CpG island in the SNU-423, SNU-398 and HUH-7 cells, whereas for HEP3B2-1-7, SNU-475 and SNU-387 cell lines the *NSUN7* 5’-end was unmethylated (Fig. 1C). Normal liver was also unmethylated (Fig. 1C). These data matched the DNA methylation profiles derived from the microarray approach (Fig. 1D). The promoter CpG island of *NSUN7* was found unmethylated in all the normal human liver tissues analyzed from the TCGA data (**Dataset S1E**). Importantly, the unmethylated liver cancer cell lines at the *NSUN7* 5’-end CpG island (HEP3B2-1-7, SNU-475 and SNU-387) expressed *NSUN7* RNA, determined by quantitative real-time PCR, furthermore the *NSUN7*-hypermethylated cells from SNU-423, SNU-398 and HUH-7 showed lack or minimal expression of the transcript (Fig. 1E). An additional link between DNA methylation and transcriptional silencing was established using the DNA demethylating agent 5-aza-2′-deoxycytidine, that restored *NSUN7* expression in the hypermethylated liver cancer cell lines (Fig. 1F).

### ***NSUN7*** Epigenetic Loss Induces Hypomethylation in the mRNA of the RNA-Binding Protein CCDC9B and Protein Downregulation

**Fig. 2:**
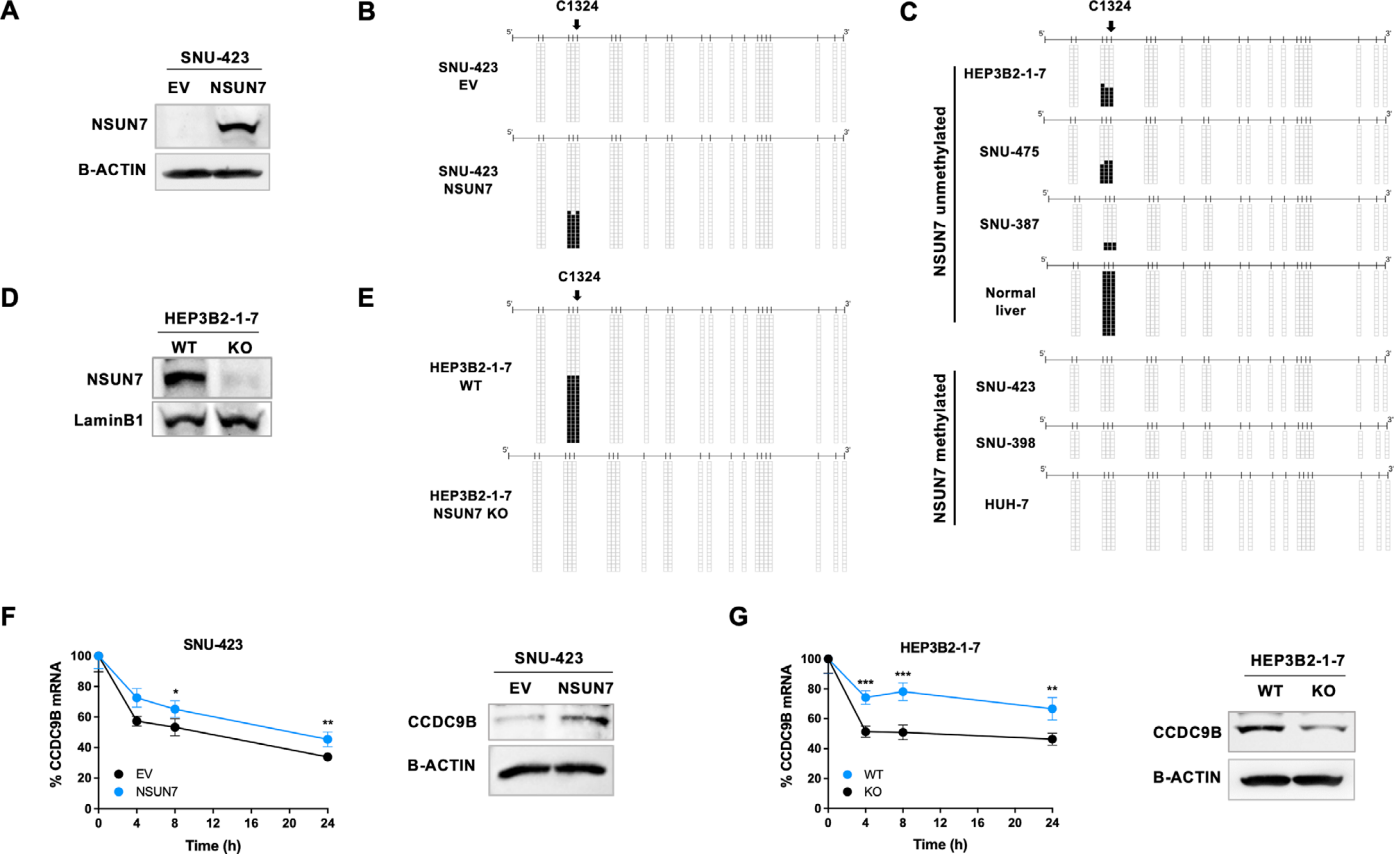
*NSUN7* epigenetic loss abrogates m^5^C methylation of the *CCDC9B* mRNA leading to diminished RNA stability and protein downregulation. (**A**) Restoration of NSUN7 protein expression by stable transduction in epigenetically-silenced SNU-423 HCC cells, analyzed by western blot. (**B**) RNA bisulfite sequencing of multiple clones of *CCDC9B* transcripts from empty vector (EV) and *NSUN7*-transfected SNU-423 cells. Cytosines are represented as short vertical lines, the bsRNAseq target C1324 is indicated by a black arrow, and presence of a methyl group is represented as a black square. (**C**) RNA bisulfite sequencing of *CCDC9B* transcripts from the HCC cell panel plus a normal liver sample, according to the basal *NSUN7*-promoter methylation status in liver cancer cell lines. (**D**) Efficient CRISPR-Cas9 mediated knockout (KO) of NSUN7 protein expression in the hypomethylated and expressing HEP3B2-1-7 HCC cell line. (**E**) RNA bisulfite sequencing of the *CCDC9B* transcript from NSUN7 WT and NSUN7 KO HEP3B2-1-7 cells. (**F**) *CCDC9B* transcript half-life analyzed by Actinomycin D chase assay (left) and CCDC9B protein levels (right) in EV and *NSUN7*-transfected SNU-423 cells. (**G**) *CCDC9B* transcript half-life analyzed by Actinomycin D chase assay (left) and CCDC9B protein levels (right) in NSUN7 WT and KO HEP3B2-1-7 cells

The targets of the 5-methylcytosine RNA methyltransferase activity of NSUN7 in human cells are unknown. For mouse cells, NSUN7 has been reported to act on the 5-methylcytosine levels of a few enhancer RNAs [25], but the sequences of these regulatory transcripts are not conserved in humans. Thus, we used a nonbiased epitranscriptomic approach to characterize likely candidate RNA transcripts modified by NSUN7. To accomplish this aim, we coupled bisulfite conversion of cellular RNA with next-generation sequencing (bsRNA-seq) [3, 6] (**Methods**), as previously described [16], to identify NSUN7-dependent modified cytosine loci in the human transcriptome. For a model, we used SNU-423, a liver cancer cell line, which displays hypermethylation-associated silencing of the *NSUN7* promoter. Herein, we generated *NSUN7*-transfected SNU-423 cells and SNU-423 cells with an empty-vector (EV) to enable comparison. Using the criteria described in **Methods**, we identified 925 candidate m^5^C sites in the transcriptome-wide mapping of *NSUN7*-transfected cells (Table S1). Upon efficient restoration of NSUN7 expression, determined by western-blot (Fig. 2A), we observed that the cytosine site in RNA that reached highest methylation levels with the most significant p-value (multiple correction adjusted P-value = 0.00001378) following *NSUN7* transfection in comparison to EV-transfected cells was the C1324 position of the mRNA of the coiled-coil domain containing 9B (*CCDC9B*) gene (Table S2). To validate this potential target, we performed next the bisulfite sequencing of multiple clones of the mRNA extracted from the empty vector and *NSUN7*-transfected SNU-423 cell line, using primers encompassing the candidate NSUN7-methylation position at the *CCDC9B* transcript. This targeted approach validated the bsRNA-seq data by showing an unmethylated C1324 position in empty-vector transfected cells and the restoration of a methylated C1324 site upon *NSUN7* transfection (Fig. 2B). Interestingly, we also observed that the two cytosines located immediately before the C1324 site, C1323 and C1322, followed the same RNA methylation profile (Fig. 2B). Most importantly, we generated an inert *NSUN7* catalytic mutant form (Fig. S1A,B) that, upon transfection in SNU-423 cells (Fig. S1C), was unable to methylate *CCDC9B* mRNA (Fig. S1D), further supporting the direct role of the enzyme in the methylation of this target.

We were able to reproduce the above described NSUN7-dependent RNA methylation patterns of the *CCDC9B* transcript in our panel of unmodified liver cancer cell lines. We found that whereas promoter unmethylated and NSUN7-expressing cell lines HEP3B2-1-7, SNU-475 and SNU-387 showed methylation of the three described mRNA sites of *CCDC9B*; the *NSUN7*-hypermethylated and non-expressing cells from SNU-423, SNU-398 and HUH-7 showed a lack of C1324, C1323 and C1322 *CCDC9B* RNA methylation (Fig. 2C). Importantly, non-tumoral liver tissue showed methylation of the three characterized cytosine sites of the *CCDC9B* mRNA (Fig. 2C). Finally, we demonstrated the role of NSUN7 in the methylation of these RNA loci in the reverse genetic engineering experiment by knocking-out the *NSUN7* gene using the CRISPR-Cas9 approach in the *NSUN7* unmethylated and expressing HEP3B2-1-7 cells. Upon efficient deletion of NSUN7 (Fig. 2D), we observed that if the wild-type cells showed C1324, C1323 and C1322 *CCDC9B* RNA methylation; the genetic abrogation of *NSUN7* induced an unmethylated status of these three cytosine sites in the *CCDC9B* transcript (Fig. 2E). Overall, all these data indicate that NSUN7 catalyzes the 5-methylcytosine methylation of the *CCDC9B* mRNA and that *NSUN7* epigenetic inactivation induces the loss of these RNA modifications in liver cancer cells.

The occurrence of 5-methylcytosine in RNAs has been proposed to be associated with transcript stability [26, 27], thus we assessed if this was the case for *CCDC9B* in our models. Using actinomycin D chase assays, we demonstrated that the transfection-mediated recovery of NSUN7 expression in the epigenetically silenced SNU-423 cell line was associated with an increase in *CCDC9B* mRNA stability (Fig. 2F) that also led to an upregulation of CCDC9B at the protein level (Fig. 2F). The transfection of the *NSUN7* mutant form did not affect the stability of the *CCDC9B* mRNA in the actinomycin assay (Fig. S1E) or the protein expression levels of CCDC9B (Fig. S1F). On the contrary, the *NSUN7* CRISPR/Cas9 deletion in the HEP3B2-1-7 cell line (unmethylated at the *NSUN7* promoter and expressing the gene) induced a decrease in *CCDC9B* transcript stability in the actinomycin D assay (Fig. 2G) that also led to a downregulation of CCDC9B at the protein level (Fig. 2G). Thus, these m^5^C demethylation events at the *CCDC9B* mRNA, that occur upon *NSUN7* epigenetic inactivation are associated with diminished expression levels of the gene targeted by the studied 5-methylcytosine RNA methyltransferase.

### Loss of CCDC9B targets the MYC-Regulator IVNS1ABP protein rendering sensitivity to Bromodomain inhibitors

**Fig. 3:**
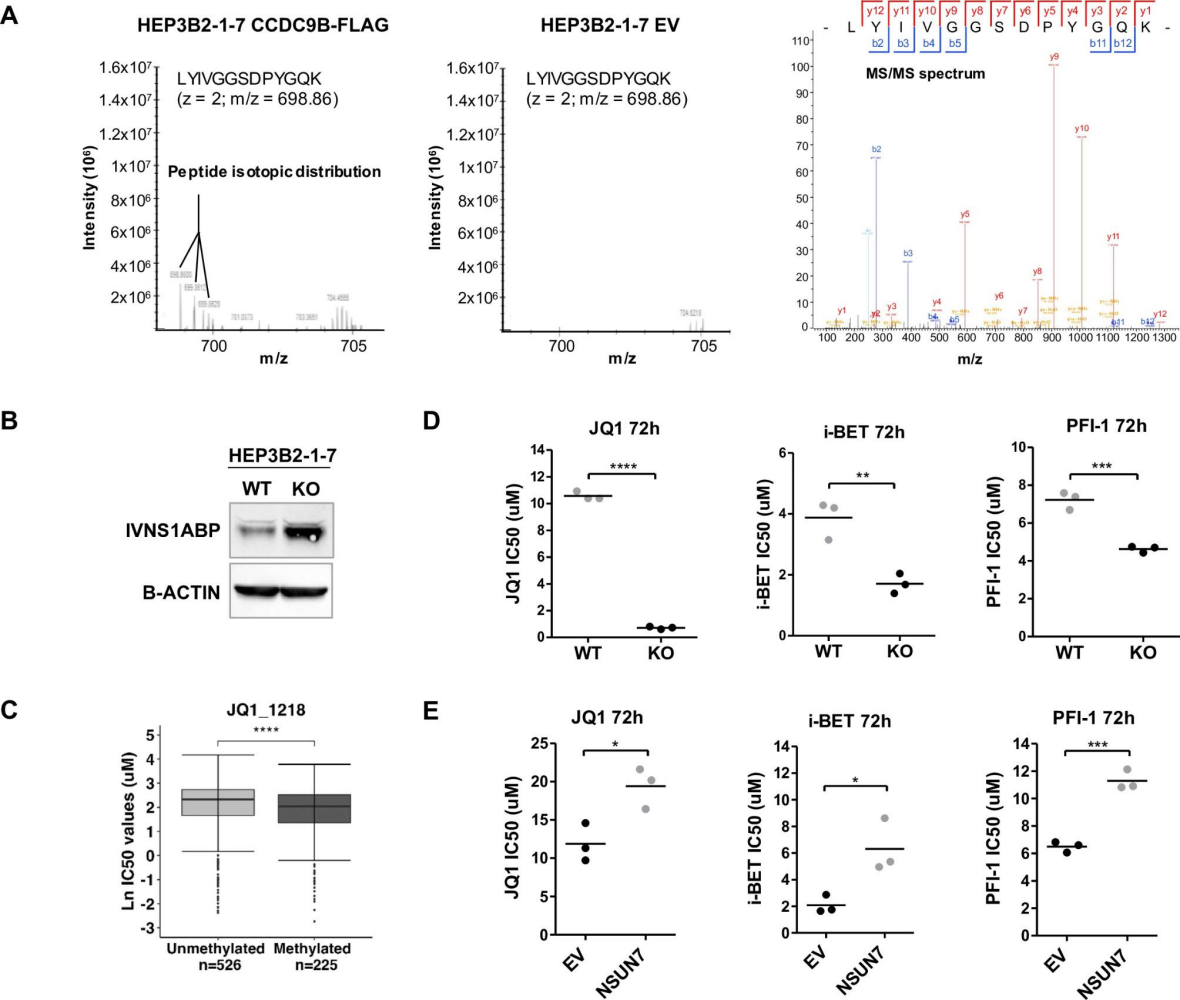
Loss of CCDC9B is associated with higher abundance of the MYC-regulator IVNS1ABP and sensitivity to bromodomain inhibitors. (**A**) MS and MS/MS spectra corresponding to the peptide LYIVGGSDPYGQK (IVNS1ABP protein) derived from the protein interactome experiments. At left, extracted Ion chromatogram showing the peptide isotopic distribution found in the *CCDC9B*-FLAG transfected HEP3B2-1-7 cells, and its absence in EV-transfected cells (middle). The identification of the peptide is shown at right (MS/MS spectrum). The b- and y-ions are depicted in blue and red, respectively. (**B**) IVNS1ABP protein levels in NSUN7 WT and KO HEP3B2-1-7 cells analyzed by western blot. (**C**) JQ1 bromodomain inhibitor IC50 values in Sanger cell lines according to *NSUN7*-promoter methylation status shows enhanced sensitivity to JQ1-mediated growth inhibition in epigenetically-silenced cells. The units in the “Y” axis are the natural logarithm (Ln) of the IC50 micromolar values. Mann–Whitney–Wilcoxon test, ****p-value < 0.0001. (**D**) IC50 determination for three bromodomain inhibitors using the SRB assay in NSUN7 WT HEP3B2-1-7 cells compared to NSUN7 KO HEP3B2-1-7 cells. (**E**) IC50 values for the three bromodomain inhibitors in *NSUN7*-transfected SNU-423 cells in comparison with EV-transfected cells. Data shown represent IC50 values obtained in biological triplicates, and p-values were calculated by a Student’s T test. *p-value < 0.05, **p-value < 0.01, ***p-value < 0.001, ****p-value < 0.0001

Very little is known about the biological function of the CCDC9B protein beyond its location in several protein and RNA remodeling complexes [28–30]. Due to the presence of the coiled-coil protein domain that could act in protein-protein interactions [31], we wondered if it could exhibit this role in our models. Thus, to look for candidate protein partners of CCDC9B that can provide further clues about its biological roles, we combined immunoprecipitation and mass spectrometry (MS). We compared immunoprecipitates from *CCDC9B*-FLAG transfected HEP3B2-1-7 cells (*NSUN7* unmethylated and expressing the gene) to immunoprecipitates of HEP3B2-1-7 cells transfected with the empty vector. Using the Human SwissProt protein database and the Significance Analysis of Interactome (SAINT) algorithm, we identified four proteins that were bound by CCDC9B: IVNS1ABP, CHTOP, PDIA4 and RCN2 (Bayesian Fold Discovery Rate < 0.05) (Table S3). As an example the MS spectra corresponding to the peptide LYIVGGSDPYGQK of the IVNS1ABP protein is shown in Fig. 3A.

Once we observed the interaction between CCDC9B and these proteins, we wondered if CCDC9B could regulate the levels of these targets by this protein-protein binding mechanism. We addressed this point, by performing global proteomics analyses by mass spectrometry in empty-vector transfected SNU-423 cells, showing *NSUN7* promoter hypermethylation-associated silencing (Fig. 1C,D,E,F), in comparison with *NSUN7*-transfected SNU-423 cells (Fig. 2A). We identified 579 proteins that had significantly different expression upon NSUN7 transfection in SNU-423 cells, 280 were downregulated and 299 upregulated (Table S4). Most importantly, among the first set, we identified IVNS1ABP, detected above as a protein partner for CCDC9B (Table S3), as one of the top-downregulated proteins upon restoration of NSUN7 expression (Table S4). Further validating these data, the opposite results were observed in the reverse model: *NSUN7* CRISPR/Cas9 deleted HEP3B2-1-7 cells showed, by western-blot, IVNS1ABP protein upregulation compared to the *NSUN7* unmethylated and expressing wild-type cells (Fig. 3B). These data support that NSUN7-mediated methylation of *CCDC9B* mRNA stabilizes this transcript allowing the CCDC9B protein interaction with IVNS1ABP and the lower abundance of this last multifunctional factor.

Interestingly with regard to cancer biology and for therapy-related facets, a role for IVNS1ABP in MYC-associated pathways has been reported [32–34] and a certain degree of association between sensitivity to bromodomain inhibitors and enhanced MYC-signaling has also been described [35–37]. Thus, we decided to interrogate how these networks and potential treatment vulnerabilities could be mediated by *NSUN7* epigenetic inactivation acting through CCDC9B loss and IVNS1ABP engagement. To assess this, we wondered if NSUN7 activity was associated with MYC expression. We observed that *NSUN7* CRISPR/Cas9 deletion in HEP3B2-1-7 cells induced the overexpression of MYC (Fig. S2A). On the reverse experiment, the restoration of NSUN7 expression by transfection in the *NSUN7* hypermethylated and silenced SNU-423 cells reduced MYC expression (Fig. S2B). In this last setting, the transfection of the inert *NSUN7* catalytic mutant form did not affect MYC expression (Fig. S2B).

We then data mined the collection of 1001 human cancer cell lines in which we had previously obtained the DNA methylation landscapes and where sensitivity to the bromodomain inhibitor JQ1, whose activity depend on MYC activation, was also available [18]. This analysis showed that *NSUN7* promoter CpG island hypermethylation was associated with increased sensitivity to JQ1 (Fig. 3C). We then moved from the *in silico* results to the wet data by demonstrating that the CRISPR/Cas9 mediated deletion of *NSUN7* in the HEP3B2-1-7 cell line (*NSUN7* unmethylated and expressing the gene) induced an increase in the sensitivity not only to JQ1 (Fig. 3D), validating the *in silico* data (Fig. 3C), but to two additional bromodomain inhibitors (PFI-1 and i-BET) [37], in comparison to the wild-type cells (Fig. 3D). Most importantly, the opposite phenotype was observed in the SNU-423 cells (showing *NSUN7* promoter hypermethylation-associated silencing) where the restoration of NSUN7 expression by transfection decreased sensitivity to the anti-growth effect of JQ1, PFI-1 and i-BET (Fig. 3E). The transfection of the *NSUN7* mutant form did not affect bromodomain inhibitor sensitivity (Fig. S2C). Thus, all this evidence suggests that NSUN7 loss, through the CCDC9B-IVNS1ABP-MYC axis described above, could render liver cancer cells more sensitive to the action of bromodomain inhibitors.

### ***NSUN7*** Epigenetic Loss Pinpoints Primary Liver Tumors with Shorter Overall Survival

**Fig. 4:**
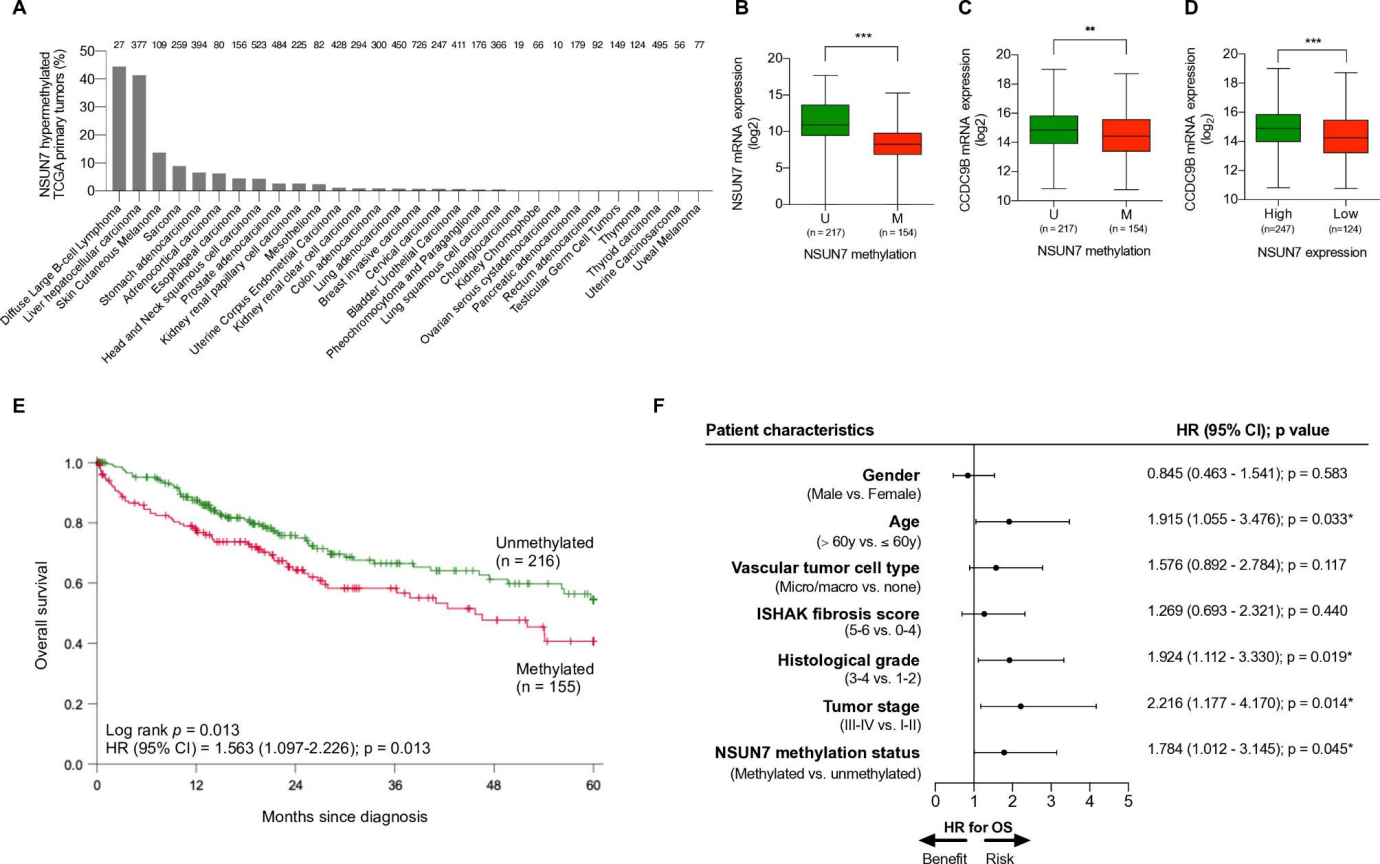
*NSUN7* epigenetic loss occurs in human primary HCC tumors in association with worse clinical outcome. (**A**) Percentage of *NSUN7* methylation in the TCGA data set of primary tumors according to cancer type. (**B**) *NSUN7* methylation is inversely correlated with *NSUN7* transcript expression in TCGA HCC tumors. (**C**) *NSUN7* methylation is associated with decreased *CCDC9B* transcript levels in primary TCGA HCC tumors. (**D**) Low expression of the *NSUN7* mRNA is associated with decreased *CCDC9B* transcript levels in primary TCGA HCC tumors. (**E**) Kaplan–Meier analysis of overall survival (OS) in the TCGA liver cancer cohort with respect to *NSUN7* methylation status. Significance of the log-rank test is shown. Results of the univariate Cox regression analysis are represented by the hazards ratio (HR) and 95% confidence interval (95% CI). (**F**) Forest plot of the multivariable Cox regression analysis for clinical outcome in the TCGA liver cohort studied for *NSUN7* methylation status taking into account different prognostic factors. P-values correspond to hazard ratios (HR), with a 95% CI, associated with OS. Co-variables with associated p-value under 0.05 were considered as independent prognostic factor (*p < 0.05, **p < 0.01, ***p < 0.001)

Once we demonstrated in liver cancer cell lines the occurrence of tumor-specific DNA methylation-associated transcriptional silencing of *NSUN7* and its downstream effects, we wondered about the presence of epigenetic inactivation of this particular 5-methylcytosine RNA methyltransferase in human primary liver tumors. Data mining of the set of primary liver tumors from The Cancer Genome Atlas (TCGA) (https://portal.gdc.cancer.gov), which were analyzed by the same DNA methylation microarray as the one used herein for our initial cancer cell line screening, showed the occurrence of *NSUN7* CpG hypermethylation in 41.4% (156 of 377) of liver tumors (Fig. 4A). Analyses of the TCGA RNA-sequencing data validated the in vitro cancer cell lines results, showing that in the primary liver tumors *NSUN7* hypermethylation was also associated with low-levels of the transcript (Student t-test, P < 0.001) (Fig. 4B). All normal liver tissues from the TCGA cohort were unmethylated at *NSUN7*, and the *NSUN7* expression levels were similar to those observed in the unmethylated cases of liver carcinoma (Fig. S3A). Interestingly, as we showed in our liver cancer cell lines models how the loss of NSUN7 was associated with lower CCDC9B stability and lower levels of this mRNA (Fig. 2F,G), we were able to recapitulate these data in the primary setting where the presence of *NSUN7* hypermethylation was associated with *CCDC9B* transcript downregulation in the TCGA cohort of liver tumors (Fig. 4C). Importantly, the association between *NSUN7* methylation and low expression of *CCDC9B* was validated in an additional independent cohort of liver tumors [21] (Fig. S3B). Low mRNA levels of *NSUN7* in both hepatocellular cancer cohorts were also associated with lower expression of *CCDC9B* (Fig. 4D, Fig. S3C). Interestingly, the presence of low levels of expression for *CCDC9B* was associated with higher levels of *MYC* mRNA in the two studied sets of liver tumors (Fig. S4A). For the TCGA cohort, expression data of a subset of proteins is available, and we found that low levels of *NSUN7* mRNA were also associated with higher levels of the MYC protein (Fig. S4B). All these data strengthen the results observed in liver cancer cell line models (Fig. S2A,B). Related to the clinicopathological context of *NSUN7* epigenetic inactivation in liver tumors, we observed that *NSUN7* hypermethylation was not associated with gender, vascular tumor cell type, ISHAK fibrosis score, histological grade and TNM stage (Table 1). However, we found that *NSUN7* epigenetic loss was more commonly observed in older patients (Fisher’s exact test, p < 0.001) (Table 1).

**Table 1:** Clinicopathological characteristics of the TCGA LIHC cohort

	NSUN7 status		
	Unmethylated (N = 221)	Methylated (N = 156)	p value*
**Gender**, n (%)			
Male	143 (64.7)	112 (71.8)	0.180
Female	78 (35.3)	44 (28.2)	
**Age**, n (%)			
≤ 60 years	125 (56.8)	55 (35.3)	< 0.001
> 60 years	95 (43.2)	101 (64.7)	
**Vascular invasion**, n (%)			
Microvascular or macrovascular	64 (34.2)	47 (35.1)	0.9056
None	123 (65.8)	87 (64.9)	
**ISHAK fibrosis score**, n (%)			
0: No fibrosis	47 (40.5)	29 (28.4)	
1,2: Portal fibrosis	16 (13.8)	15 (14.7)	
3,4: Fibrous septa	15 (12.9)	15 (14.7)	0.449
5: Nodular formation and incomplete cirrhosis	4 (3.4)	5 (4.9)	
6: Established cirrhosis	34 (29.3)	38 (37.3)	
**Histological grade**, n (%)			
Grade 1	34 (15.4)	21 (13.9)	
Grade 2	104 (47.1)	76 (50.3)	0.878
Grade 3	76 (34.4)	48 (31.8)	
Grade 4	7 (3.2)	6 (4.0)	
**TNM stage**, n (%)			
Stage I	99 (48.1)	76 (51.7)	
Stage II	51 (24.8)	36 (24.5)	0.293
Stage III	51 (24.8)	35 (23.8)	
Stage IV	5 (2.4)	0 (0.0)	

*p-values were calculated using Fisher’s exact test or Chi-Square test for dichotomous or categorical variables, respectively. p-values under 0.05 represent statistical significant association between co-variables. Available data is shown

We also interrogated whether the presence of *NSUN7* hypermethylation exhibited any prognostic value in liver tumors. In this regard, we found that *NSUN7* epigenetic inactivation was associated with reduced overall survival (OS) in the TCGA cohort (log-rank P = 0.013; hazard ratio (HR) = 1.563, 95% CI = 1.097–2.226) (Fig. 4E). *NSUN7* hypermethylation was a better predictor of OS than *NSUN7* expression that can be affected by many factors beyond epigenetic inactivation. The low levels of *NSUN7* were only associated with shorter survival in the non-vascular tumor type [65.4% of the cases (log-rank P = 0.021; HR = 1.852, 95% CI = 1.089–3.149] (Fig. S5) among the examined clinicopathological characteristics (Table 1). Most importantly, to assess the value of *NSUN7* methylation as a potential independent biomarker, we performed multivariate analysis for *NSUN7* methylation and the available clinical parameters (gender, age, vascular tumor cell type, ISHAK fibrosis score, histological grade and TNM stage) in the TCGA liver cancer cohort. Remarkably, the multivariate Cox regression analysis showed that *NSUN7* hypermethylation was an independent predictor of overall survival (HR = 1.784; 95% CI = 1.012–3.145; P = 0.045) (Fig. 4F).

Finally, we wondered if our subset of cases with *NSUN7* hypermethylation were enriched in any particular subclass of liver tumors. Using a recently revised immunogenomic classification of liver tumors [38], we found that TCGA HCC cases hypermethylated at *NSUN7* were significantly depleted in the immune active subclass (Fisher’s exact test, P = 0.018), but enriched for the immune-like signature (Fisher’s exact test, P = 0.002). Liver tumors from both subclasses present an enrichment in the PD-1 and interferon signaling pathway and high expression of checkpoint molecules (such as CTLA4, PD-1 and PD-L1) [38], all of them biomarkers associated with response to immunotherapy, but the immune active subclass is associated with increased overall survival and the immune-like signature is not [38]. Importantly using the Chiang molecular classification of HCC [39], we observed that TCGA liver tumors hypermethylated at *NSUN7* were significantly enriched in the CTNNB1 subclass (Fisher’s exact test, P = 0.011) and it has been recently proposed that β-catenin activation in HCC promotes resistance to anti-PD-1 therapy [40]. Thus, the described analyses warrant further investigation into a role of NSUN7 in immunotherapy response.

## Discussion

The development and progression of human tumors were initially associated with the acquisition of chromosomic and genetic defects, but in the last two decades the occurrence of epigenetic alterations, affecting DNA methylation, histone modifications or chromatin organization, also have been demonstrated to contribute to the natural history of cancer. Another layer of gene regulation which is also gaining momentum in cancer biology, is the presence of disrupted patterns of RNA modifications, the denominated epitranscriptome. In this regard, beyond shifts in the epitranscriptomic marks themselves, there is a growing body of evidence that indicates that dysregulation of the RNA modification proteins, of which many of their targets are central to hallmarks of cancer cells, such as proliferation, self-renewal and DNA repair are key players in the etiology of cancer [1, 2]. Although a majority of the scientific literature in this area deals with the m^6^A RNA mark, other chemical modifications of RNA such as m^5^C [10–12] and its associated proteins [13] are also emerging as aberrantly involved in human tumorigenesis. In this regard, roles of m^5^C in determining the functionality of structural noncoding RNAs such as tRNAs have now been widely reported [15, 41, 42], but the action of m^5^C RNA methyltransferases in mRNA is not so well characterized, even less in the context of transformed cells. Herein, we have demonstrated that a previously poorly characterized m^5^C RNA methyltransferase, NSUN7, undergoes cancer-specific epigenetic transcriptional silencing in different tumor types. Focusing in liver cancer, we have been able to show that this enzyme exerts its m^5^C RNA methyltransferase activity on mRNA, characterizing the transcript of coiled-coil domain containing 9B (*CCDC9B*) gene as a relevant target in hepatic cancer. We have also shown that the addition of the m^5^C modification in *CCDC9B* mRNA stabilizes the molecule, whereas the *NSUN7* mediated epigenetic inactivation in liver cancer decreases *CCDC9B* mRNA half-life. In this last case, the diminished *CCDC9B* mRNA levels are associated with the upregulation of the IVNS1ABP protein, a MYC regulator [32–34]. These results constitute some of the first evidence tying together the imbalance of m^5^C modification in mRNA with a defined biological activity (RNA instability) of the mark in a transcript in the cancer context.

Interestingly, although most of the m^5^C sites in human mRNA seem to be deposited by two other 5-methylcytosine RNA methyltransferases, NSUN2 and NSUN6 [43, 44], we have observed additional potential targets for NSUN7-mediated RNA methylation in our bsRNA-seq approach. In addition, it is possible that other candidate cytosine sites targeted by NSUN7 were missed in our experimental pipeline due to the used bioinformatic cut-offs and technical limitations such a moderate sequencing depth or low stoichiometry of the enzymatic reaction. Both aspects are worth of further investigation.

Beyond the basic significance of unveiling a new functionality of m^5^C in mRNA, our results might have a relevant impact in the understanding and management of liver tumors. Hepatic malignancies are one of the most common neoplasms globally, unfortunately, they are also often associated with poor patient clinical outcome and survival [24]. Herein, we have shown that *NSUN7* promoter hypermethylation-associated silencing can be detected in an important fraction of primary liver tumors and that the presence of *NSUN7* epigenetic inactivation is associated with reduced overall survival. Importantly, our findings may help pave the way for patients to access new therapies and enrich patients which are sensitive to other newly introduced agents, based on *NSUN7* status. Related to the first scenario, our *in silico* and experimental data in the original liver cancer cells and upon genetic engineering of *NSUN7* (CRISPR/Cas9 mediated-deletion and recovery of activity by transfection) indicates that NSUN7 loss sensitizes the transformed cells to the antiproliferative effects of bromodomain inhibitors. This family of epigenetic drugs is intensively assessed in ongoing cancer clinical trials and those liver tumors harboring *NSUN7* epigenetic inactivation might constitute an unexpected niche of patients where these compounds could possess efficacy. Secondly, our analyses of primary liver tumors showed an overrepresentation of cases with an NSUN7 unmethylated promoter in those categorized as displaying an immune active signature, characterized by an enrichment in the PD-1 and interferon signaling pathway with high expression of checkpoint molecules (such as CTLA4, PD-1 and PD-L1) and associated with improved overall survival [38]. Thus, this subset of cases could be more amenable to the use of immune checkpoint inhibitors, a line of treatment recently introduced for liver cancer [45], but so far without any clear biomarker predictive to the clinical response for these agents. The above two examples of candidate tailored treatments linked to NSUN7 epigenetic status might be nice additions to include in the portfolio of biomarkers for ongoing clinical trials for this devastating tumor type.

## Conclusions

In conclusion, we show that the m^5^C RNA methyltransferase NSUN7 undergoes promoter hypermethylation-associated silencing in human cancer. Our focus in liver models unveils that the enzyme adds the m^5^C chemical mark at the *CCDC9B* mRNA stabilizing the transcript. This equilibrium is broken down in liver tumors carrying *NSUN7* epigenetic silencing, causing a cascade of CCDC9B degradation and upregulation of its herein identified protein partner IVNS1ABP. The enhanced levels of this last protein, a MYC-regulator, renders these cells more sensitive to the antigrowth effect of bromodomain inhibitors. In the clinical setting, *NSUN7* epigenetic inactivation is associated with poor outcome, but the enrichment of *NSUN7* unmethylated cases in the immune active subclass could highlight those patients that yield a greater clinical response through the use of immune checkpoint inhibitors.

## Electronic supplementary material

Below is the link to the electronic supplementary material.

Supplementary Material 1

Supplementary Material 2

Supplementary Material 3

Supplementary Material 4

Supplementary Material 5

Supplementary Material 6

Supplementary Material 7

Supplementary Material 8

Supplementary Material 9

Supplementary Material 10

Supplementary Material 11

## Data Availability

All the obtained bsRNA-seq data have been deposited at the NIH submission portal (https://submit.ncbi.nlm.nih.gov/) with the following link: BioProject: PRJNA904551 (https://www.ncbi.nlm.nih.gov/bioproject/PRJNA904551). IP-MS/MS proteomics data have been deposited at the ProteomeXchange via the PRIDE database partner repository with the dataset identifier PXD038422 (https://www.ebi.ac.uk/pride/archive/projects/PXD038422). TMT MS proteomics data have been deposited at the ProteomeXchange via the PRIDE database partner repository with the dataset identifier PXD038418 (https://www.ebi.ac.uk/pride/archive/projects/PXD038418). The used bioinformatic scripts are available at GitHub for m^5^C site calling (https://github.com/YangLab/bsRNA-seq-m5C), the interactome (https://github.com/mesteller-bioinfolab/NSUN7) and the proteome (https://github.com/mesteller-bioinfolab/NSUN7) analyses. All other datasets used and/or analyzed during the current study are available from the corresponding author on reasonable request.
