# Supplementary material for: Epigenetic inactivation of the 5-methylcytosine RNA methyltransferase NSUN7 is associated with clinical outcome and therapeutic vulnerability in liver cancer
Source: Mol Cancer. 2023 May 12;22:83. doi: 10.1186/s12943-023-01785-z (PMC10176850; doi:10.1186/s12943-023-01785-z)
Supplement: Supplementary file 1 — Supplementary Material 1 [file 12943_2023_1785_MOESM1_ESM.docx]

**Key Resources Table**

| **ANTIBODIES** | | |
| --- | --- | --- |
| **Name** | **Supplier** | **Reference** |
| Rabbit polyclonal anti-NSUN7 | Biorbyt | orb258175 |
| Rabbit polyclonal anti-CCDC9B | Novus Biologicals | NBP1-93764 |
| Rabbit polyclonal anti-IVNS1ABP | Proteintech | 14741-1-AP |
| Monoclonal anti-β-Actin | Sigma | A3854 |
| Rabbit polyclonal anti-  Lamin B1 | Abcam | ab16048 |
| Rabbit monoclonal anti-c-Myc | Cell signaling | #5605 |

| **OLIGONUCLEOTIDES** | |
| --- | --- |
| **Cloning** | |
| **Name** | **Sequence (5’-3’)** |
| NSUN7 Cloning Fw | AAAAAAAACTCGAGCCGCCACCATGCTGAATT |
| NSUN7 Cloning Rv | AAAAAAAAGCGGCCGCTCAAAGCCATCGCCGAGGAGGCCTGAGTAGGG |
| NSUN7 MUT PCR  phusion Fw | TTACTGCACAGTGTCAGTTTTTCCAGAAGAAAATG  AAGCTGTTGT |
| NSUN7 MUT PCR  phusion Rv | GGAAAAACTGACACTGTGCAGTAAACAACTGCTT  GAGCTTTAGTAAATT |
| NSUN7 gRNA#1 Fw | CACCGCTACCTCGTTGTTCAGGAC |
| NSUN7 gRNA#1 Rv | AAACGTCCTGAACAACGAGGTAGC |
| NSUN7 gRNA#2 Fw | CACCGCACTGGAATTTCAAGACCTT |
| NSUN7 gRNA#2 Rv | AAACAAGGTCTTGAAATTCCAGTGC |
| NSUN7 gRNA#3 Fw | CACCGCTCCTTGAGAGTGATCTTTA |
| NSUN7 gRNA#3 Rv | AAACTAAAGATCACTCTCAAGGAGC |
| CCDC9B Cloning Fw | TTTTTTTTGAATTCGCCGCCACCATGCACTCGGCTGGAACTCCCAGAGCC |
| CCDC9B Cloning Rv | TTTTTTTTGGATCCTCACTTGTCGTCGTCGTCCTTGTAGTCGCCGCTGCCGCATCTTCCTGCCGGGCCAGGGC |

| **DNA bisulfite sequencing** | |
| --- | --- |
| **Name** | **Sequence (5’-3’)** |
| NSUN7 BSP Fw | TTTTAGGAATTTGTGAGTTAAAATT |
| NSUN7 BSP Rv | TCAAATACAACCACTCCCAC |

| **RNA bisulfite sequencing** | |
| --- | --- |
| **Name** | **Sequence (5’-3’)** |
| CCDC9B BS Fw | CTGGAAGGTCAGGAGGGAAG |
| CCDC9B BS Rv | GGAAGCCACTGACTCCCC |

| **Real-time qPCR** | |
| --- | --- |
| **Name** | **Sequence (5’-3’)** |
| NSUN7 qPCR Fw | TCAAACTCGTGTCCTTTCTGA |
| NSUN7 qPCR Rv | GGAGGCCCTTAGTTCCTGTT |
| CCDC9B qPCR Fw | CTGGAAGGTCAGGAGGGAAG |
| CCDC9B qPCR Rv | GGAAGCCACTGACTCCCC |
| GAPDH qPCR Fw | TGCACCACCAACTGCTTAGC |
| GAPDH qPCR Rv | GGCATGGACTGTGGTCATGAG |
