# Supplementary figures and images for: Epigenetic inactivation of the 5-methylcytosine RNA methyltransferase NSUN7 is associated with clinical outcome and therapeutic vulnerability in liver cancer
Source: Mol Cancer. 2023 May 12;22:83. doi: 10.1186/s12943-023-01785-z (PMC10176850; doi:10.1186/s12943-023-01785-z)

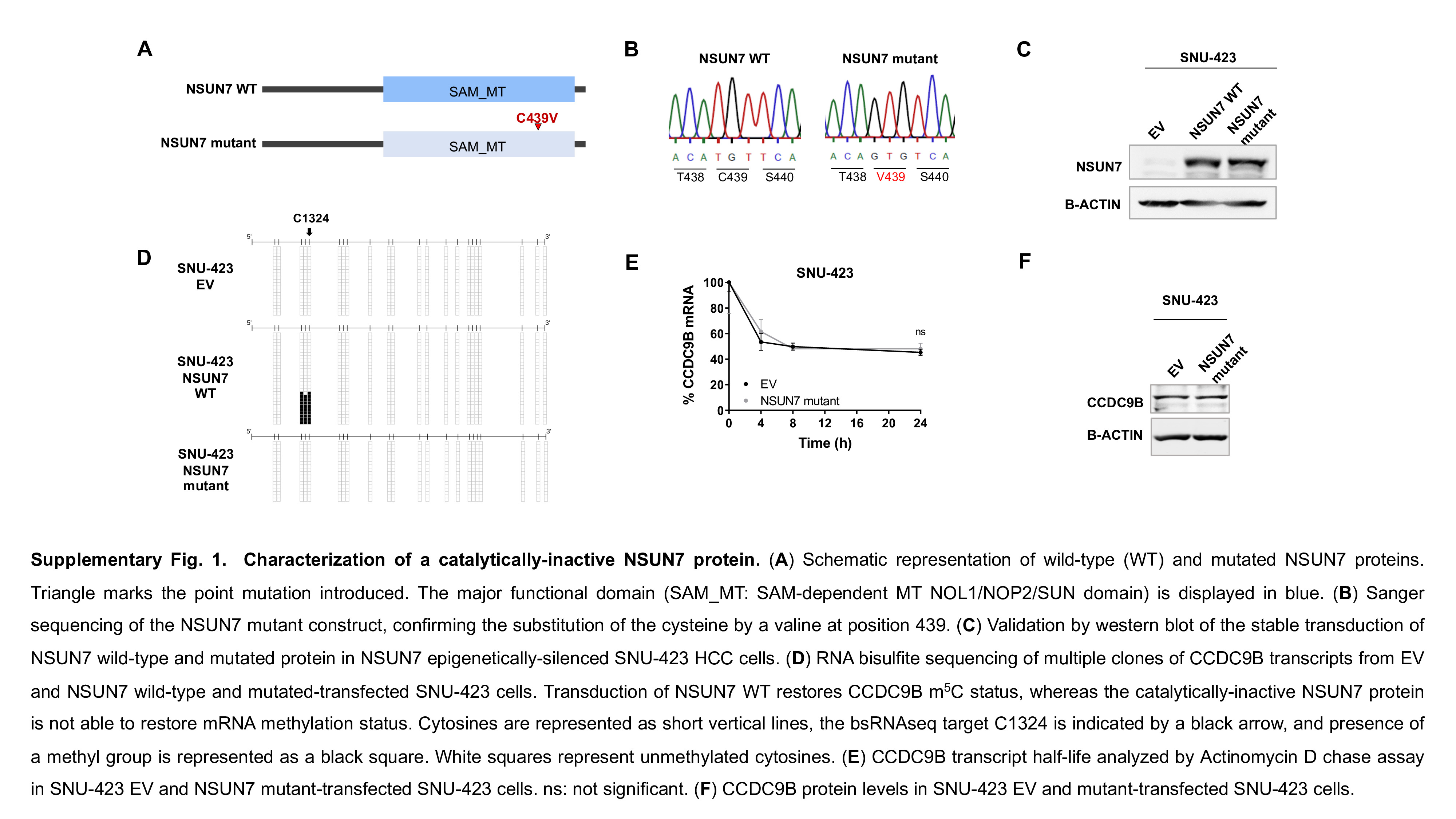

Supplement: Supplementary file 3 — Supplementary Material 3 [file 12943_2023_1785_MOESM3_ESM.jpeg]

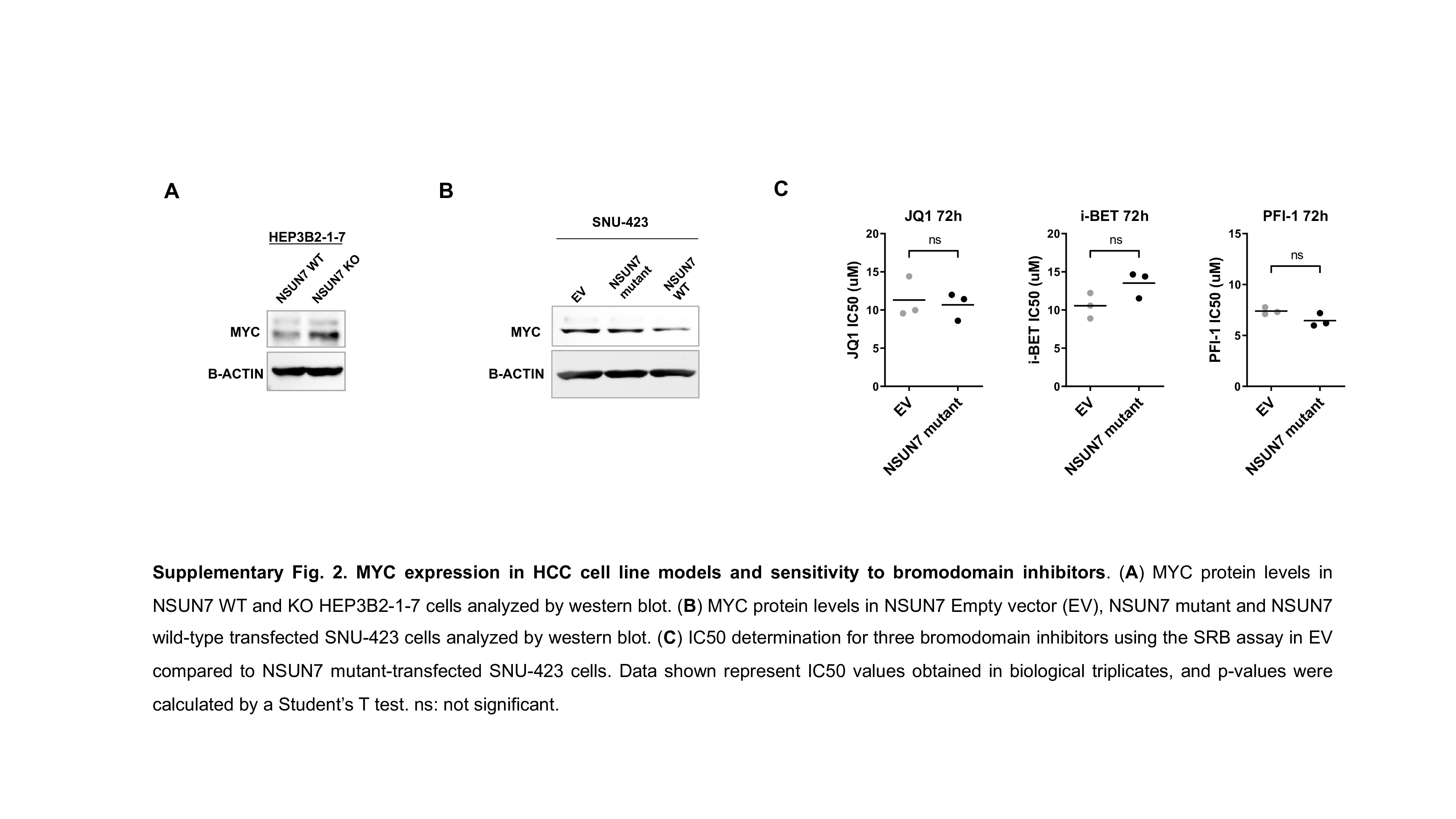

Supplement: Supplementary file 4 — Supplementary Material 4 [file 12943_2023_1785_MOESM4_ESM.jpeg]

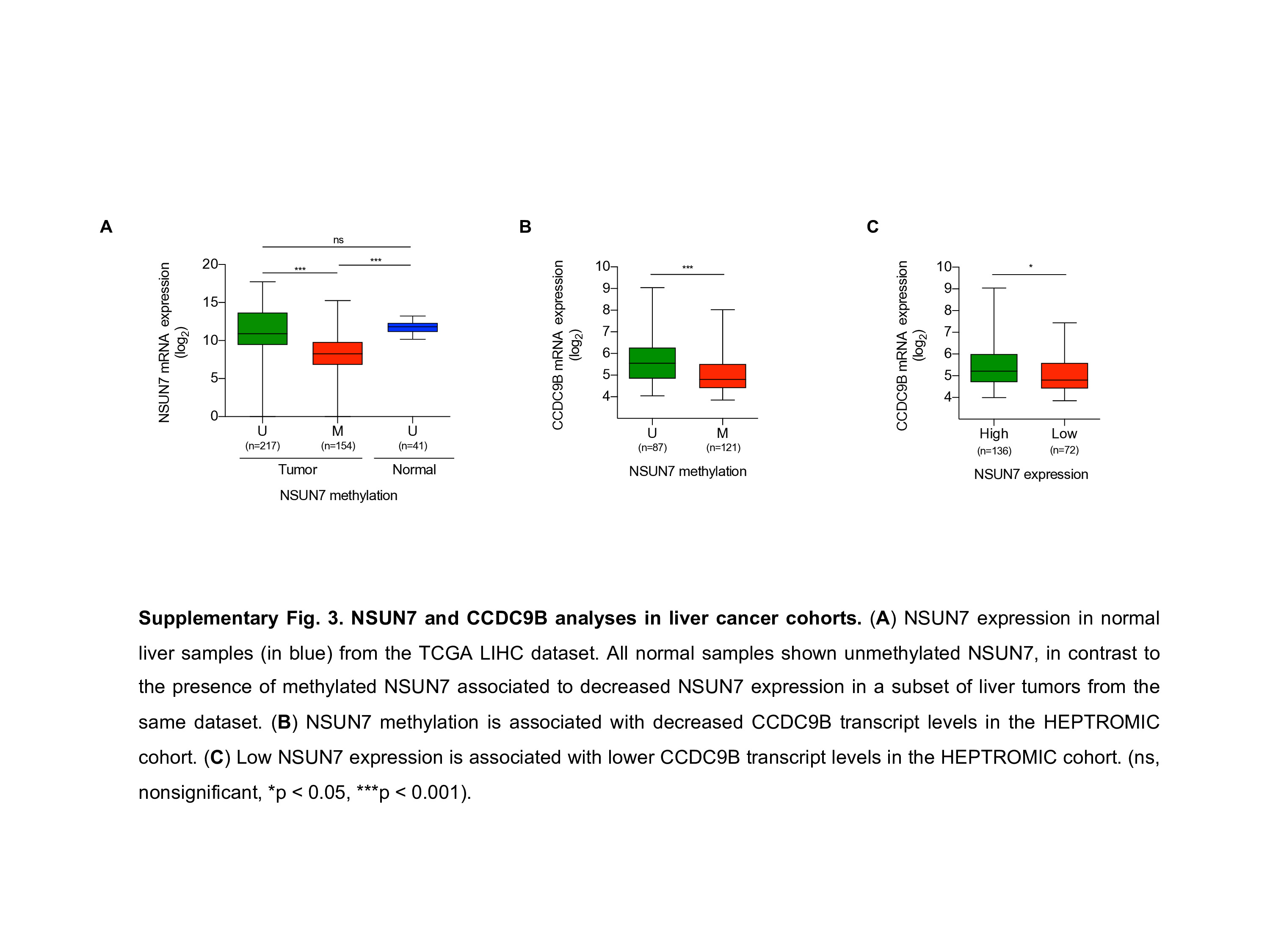

Supplement: Supplementary file 5 — Supplementary Material 5 [file 12943_2023_1785_MOESM5_ESM.jpeg]

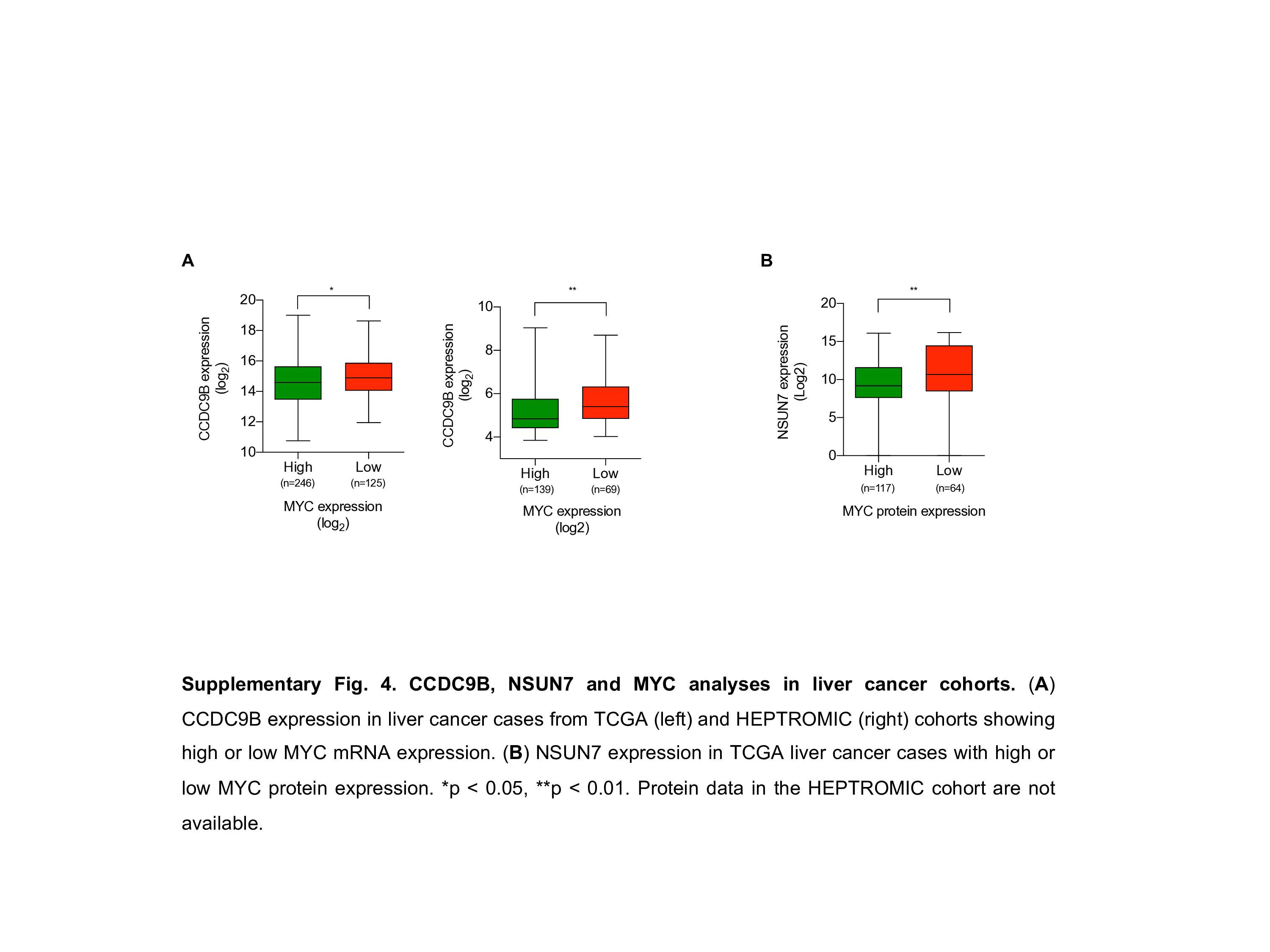

Supplement: Supplementary file 6 — Supplementary Material 6 [file 12943_2023_1785_MOESM6_ESM.jpeg]

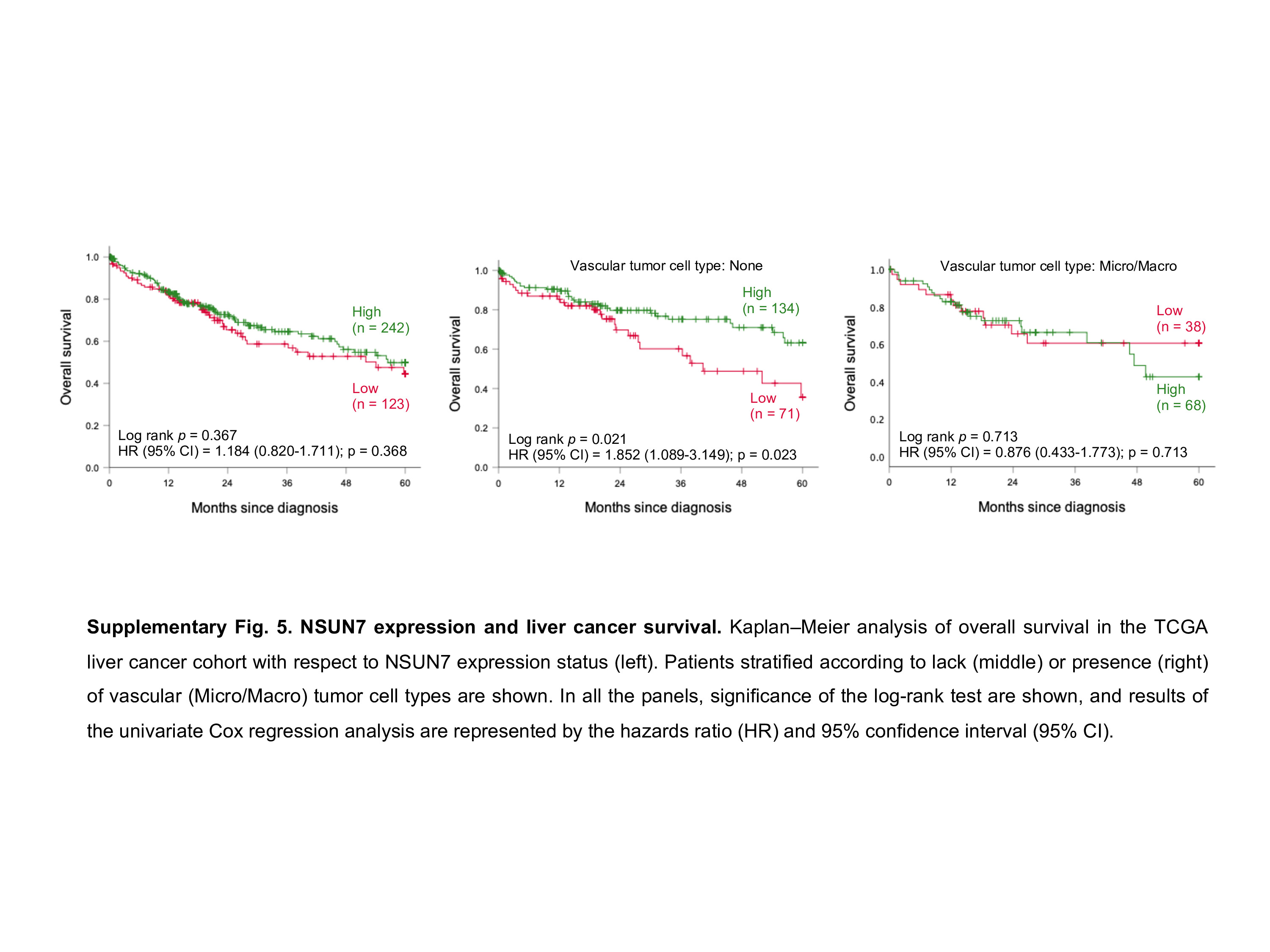

Supplement: Supplementary file 7 — Supplementary Material 7 [file 12943_2023_1785_MOESM7_ESM.jpeg]
